# Coinheritance of *HNF1A* and glucokinase variants in maturity-onset diabetes of the young

**DOI:** 10.1530/EDM-23-0100

**Published:** 2024-08-01

**Authors:** Daisuke Watanabe, Hideaki Yagasaki, Hiromune Narusawa, Takeshi Inukai

**Affiliations:** 1 Department of Pediatrics, Faculty of Medicine, University of Yamanashi, Yamanashi, Japan

**Keywords:** Paediatric, Female, Asian - Japanese, Japan, Pancreas, Diabetes, Genetics and mutation, Paediatric endocrinology, Insight into disease pathogenesis or mechanism of therapy, August, 2024

## Abstract

**Summary:**

Maturity-onset diabetes of the young (MODY) is a group of monogenic forms of diabetes mellitus characterized by early-onset diabetes with dominant inheritance of beta-cell dysfunction. There are few reports of the coinheritance of glucokinase (*GCK*) and hepatocyte nuclear factor 1 alpha gene (*HNF1A*) variants underlying MODY in patients. Herein, we describe a case involving combinations of monoallelic *GCK* and *HNF1A* variants associated with MODY. A 10-year-old Japanese girl with a three-generation family history of diabetes without obesity showed high levels of urinary glucose during a school screening test. Her glucose metabolism profile revealed 124 mg/dL of fasting glucose, 6.9% glycated hemoglobin (HbA1c), and 2.78 ng/mL of C-peptide immunoreactivity levels. In a 75-g oral glucose tolerance test, her base glucose, peak glucose, insulin resistance, and homeostasis model assessment of beta cell function levels were 124 mg/dL, 210 mg/dL (120 min), 1.71, and 33%, respectively. Based on the clinical phenotype of GCK-MODY, alimentary and exercise therapy without oral hypoglycemic agents were used to maintain her fasting glucose and HbA1c levels. We explored the coinheritance of MODY with *GCK* and *HNF1A* variants in this and past cases and found that careful clinical follow-up is required to firmly establish phenotypic features. Moreover, the accumulation of data on genetically confirmed MODY associated with the coinheritance of *GCK* and *HNF1A* variants will be useful for understanding genotype–phenotype correlations.

**Learning points:**

- MODY is a group of monogenic forms of diabetes mellitus characterized by early-onset diabetes with the dominant inheritance of beta-cell dysfunction.
- MODY2 and MODY3 caused by heterozygous loss-of-function variants in the glucokinase (*GCK*) and hepatocyte nuclear factor 1 alpha (*HNF1A*) genes, respectively, are the most common forms of the disease.
- Few cases of MODY have previously been reported as being associated with the coinheritance of *GCK* and *HNF1A* variants.
- Careful clinical follow-up is required to firmly establish phenotypic features in the coinheritance of MODY with *GCK* and *HNF1A* variants.
- The accumulation of data on genetically confirmed MODY associated with the coinheritance of *GCK* and *HNF1A* variants will be useful for understanding genotype–phenotype correlations.

## Background

Maturity-onset diabetes of the young (MODY) is a group of monogenic forms of diabetes mellitus characterized by early-onset diabetes with dominant inheritance of beta-cell dysfunction. Additional features of MODY include negative pancreatic autoantibodies and persistently detectable C-peptide levels, which differentiate it from type 1 diabetes and non-obesity and type 2 diabetes. To date, 14 different causative genes have been proposed for MODY. MODY2 and MODY3 caused by heterozygous loss-of-function variants in the glucokinase (*GCK*) and hepatocyte nuclear factor 1 alpha (*HNF1A*) genes, respectively, are the most common forms of the disease. Clinical manifestations of MODY2 include a moderate increase in fasting glycemia, typically from birth. Diabetes-associated complications are unlikely in patients with MODY2, and they rarely require pharmacological treatment. MODY3 is usually diagnosed in adolescence or early adulthood, requires pharmacological treatment, and often leads to late-onset microvascular complications. Hundreds of variants have been identified in *GCK* and *HNF1A* (1).

Typically, only one gene is thought to be causative of MODY, and a few cases of MODY have been reported to be associated with the coinheritance of *GCK* and *HNF1A* variants (1, 2, 3, 4, 5). Data on the clinical follow-up of individuals with the coinheritance of *GCK* and *HNF1A* in MODY are scarce. In addition, the coinheritance of *GCK* and *HNF1A* variants was found to be extremely rare in a recent study of cancer multi-gene panel testing (6, 7). Herein, we report a case of MODY with coinheritance of monoallelic variants in *GCK* and *HNF1A*.

## Case presentation

**Figure 1:**
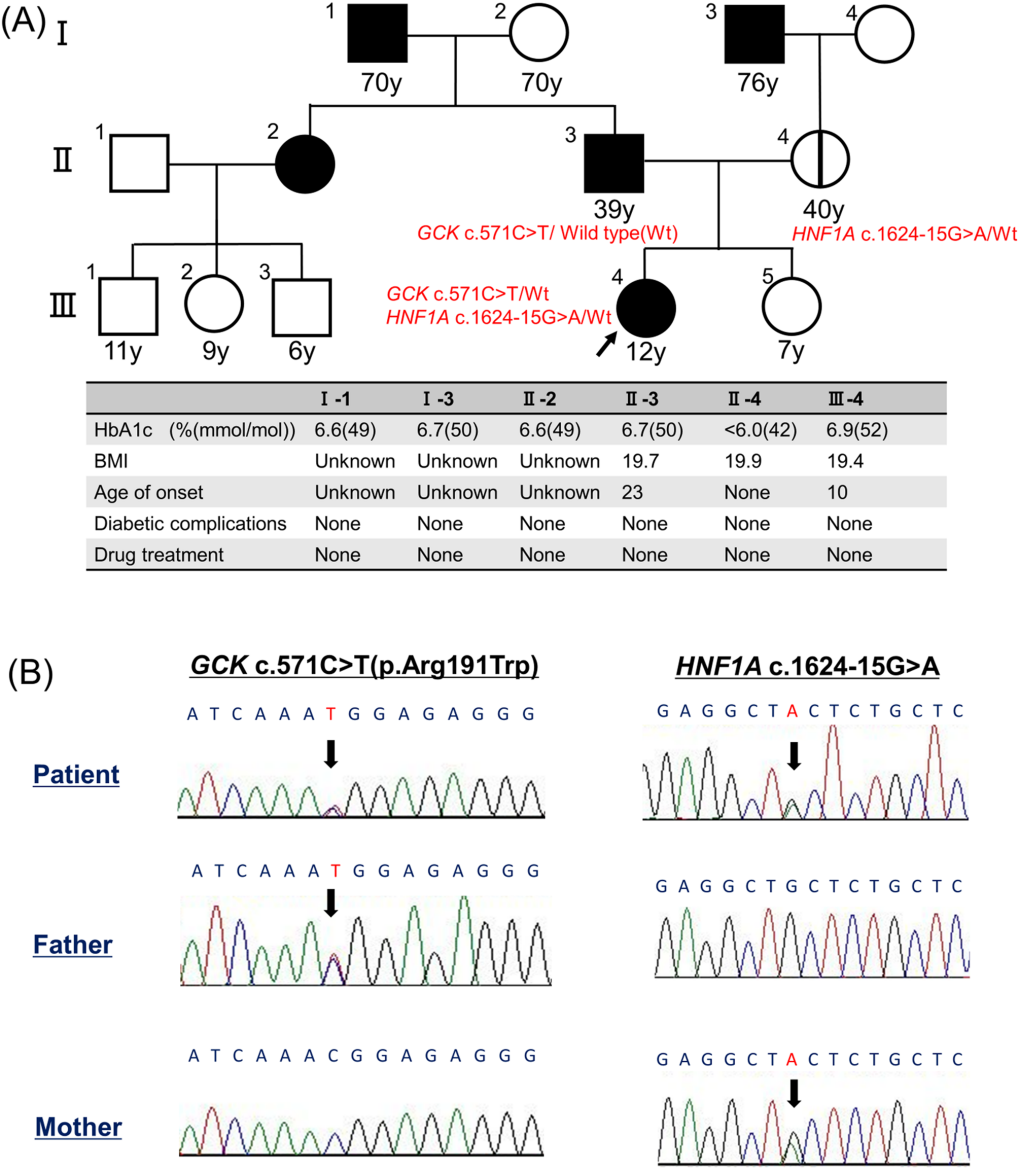
Family pedigree and sequence analysis of *GCK* and *HNF1A* in the patient and parents. A) In the family pedigree, her father was diagnosed with diabetes at the age of 23 years. On the paternal side, the patient’s father, grandfather, and aunt were diagnosed with diabetes. On the maternal side, the patient's grandfather had diabetes, but her mother and grandmother have not been diagnosed with diabetes. All family members have not needed oral hypoglycemic drugs and have been treated with diabetes dietary and exercise therapy, resulting in no diabetic complications. B) The patient harbored compound heterozygous variants *GCK* c.571C>T and *HNF1A* c.1624-15G>A. The patient’s father and mother harbored monoallelic variants *GCK* c.571C>T and *HNF1A* c.1624-15G>A, respectively.

The patient was a 10-year-old Japanese girl born with a weight of 3110 g at 40 weeks of gestation. High levels of urinary glucose were first detected during a school screening examination at the age of 10. She had a three-generation family history of diabetes (Fig. 1A). On her paternal side, her father, grandfather, and aunt had been diagnosed with diabetes but did not take oral hypoglycemic drugs. Her father was diagnosed with diabetes at the age of 23 years. On her maternal side, her grandfather had diabetes, but her mother and grandmother have not been diagnosed with diabetes. She was non-obese (height: 141.4 cm; weight: 38.8 kg), and no acanthosis nigricans was observed at the back of her neck.

## Investigation

Her glucose metabolism profile revealed 124 mg/dL (6.9 mmol/L) of fasting glucose, 150 mg/dL (8.3 mmol/L) of casual glucose, 6.9% (52 mmol/mol) glycated hemoglobin (HbA1c), 18.1% glycoalbumin, 18.9 µIU/mL of fasting immunoreactive insulin, and 2.78 ng/mL of fasting C-peptide. She exhibited negative results for anti-glutamic acid decarboxylase and anti-islet antigen-2 antibodies. In the 75 g oral glucose tolerance test (OGTT), her base glucose, peak glucose, homeostatic model assessment (HOMA)-R for insulin resistance, and insulinogic index and HOMA-β for beta cell function levels were 124 mg/dL (6.9 mmol/L), 210 mg/dL (11.7 mmol/L) (120 min), 1.71 (normal range (NR): < 2.5), 1.08 (NR: > 0.8) and 33 (NR: 40–80), respectively.

After obtaining written informed consent from her parents, a genetic analysis was conducted to evaluate glucometabolic disorders using a next-generation sequencing panel consisting of 17 genes commonly mutated in monogenic diabetes (*ABCC8, GATA6, GCK, GLUD1, HNF1A, HNF1B, HNF4A, INS, INSR, KCNJ11, NEUROD1, PDX1, AIRE, FOXP3, HADH, KLF11,* and *WFS1*). The sequenced reads were aligned to the human reference genome (GRCh38/hg38). All variants were screened according to the location, frequency, and type of variants. We applied the following criteria to select the variants: a minor allele frequency (MAF) < 0.01 in the database; located within exons or splicing regions; and missense, nonsense, and frameshift mutations, but not synonymous variants. This monogenic diabetes panel analysis identified chr7: 44149977 G>A, *GCK* (NM_000162.5: c.571C>T, p.Arg191Trp) and chr12: 120999468 G>A, *HNF1A* (NM_000545.8: c.1624-15G>A| NM_001306179.2: c.1630G>A, p.Ala544Thr) in the patient. We could not identify any significant variants in the other genes we searched. Her father carried the *GCK* monoallelic variant, and her mother carried the *HNF1A* monoallelic variant (Fig. 1b).

## Treatment

She received exercise and alimentary therapy, with no oral administration of hypoglycemic agents.

## Outcome and follow-up

The* GCK* p.Arg191Trp is classified as pathogenic (VCV000426122.21) in the ClinVar database (https://www.ncbi.nlm.nih.gov/clinvar/) with a minor allele frequency of < 0.01. *HNF1A* c.1624-15G>A is classified as having conflicting interpretations of pathogenicity (VCV000036806.6) in the ClinVar database. The minor allele frequencies of *HNF1A* c.1624-15G>A are 0.0002 and 0.0053 in the 1000 Genomes Project database (https://www.internationalgenome.org/1000-genomes-browsers) and the 4.7K Japan allele and genotype frequency panel (https://jmorp.megabank.tohoku.ac.jp/), respectively. *In silico* analysis predicted *HNF1A* p.Ala544Thr, a minor transcript with an amino acid change, to be benign (0.001) by Polyphen-2 (http://genetics.bwh.harvard.edu/pph2/index.shtml), tolerated (0.525) by SIFT (https://sift.bii.a-star.edu.sg/), and possibly pathogenic (0.229) by M-CAP (http://bejerano.stanford.edu/MCAP/) prediction tools. At her 2-year follow-up, casual glucose and HbA1c levels had been maintained near 150 mg/dL (8.3 mmol/L) and 6.5% (48 mmol/mol), respectively.

## Discussion

This report describes a case of MODY with a confirmed coinheritance of monoallelic variants of *GCK* and *HNF1A*. Our observation points to the notion that careful clinical follow-up is required to firmly establish phenotypic features in the coinheritance of MODY with *GCK* and *HNF1A* variants.

A comparison of clinical features previously reported patient and currently reported by one of the coinheritance of MODY with *GCK* and *HNF1A* variants is tabulated. We identified nine cases reported in five studies (1, 2, 3, 4, 5) (Table 1). There were no significant sex differences among the patients (males, 5; females, 3; unknown, 1). Several of these patients were diagnosed at a young age with mildly elevated glucose and HbA1c levels and persistently detectable C-peptide levels (2, 3, 4, 5), closely resembling MODY2 in that the patient’s diabetes was well controlled with exercise and dietary therapy, similar to our patient. In contrast, two adult patients had received oral administration of drugs (1, 2), resulting in a metabolic phenotype that was not entirely consistent with the *GCK* variant. Furthermore, although OGTT results were not available in the previously reported patient, our patient had high glucose levels at > 120 min, which is not a typical MODY2 phenotype; a characteristic feature of MODY3 in OGTT results is usually peak glucose levels at > 120 min (8). Careful clinical follow-up is needed to determine the phenotype, especially in pediatric patients, of the coinheritance of MODY involving *GCK* and *HNF1A* variants, which may reflect having some combined effect on glucose metabolism.

**Table 1:** Current and previous *GCK* and *HNF1A* variants in maturity-onset diabetes of the young.

Study*/subject	*GCK* variant	*HNF1A* variant	Age at diagnosis (years)	Sex	Glucose		HbA1c		CPR (ng/mL)	OGTT	Drug therapy	Phenotype
					mg/dL	mmol/L	%	mmol/L				
1												
Proband	p.Tyr61Ter	c.-154_-160dup	20	M	123	6.8	6.0	42	2.0	NA	None	IGT
Father	p.Tyr61Ter	c.-154_-160dup	34	M	124	6.9	6.6	49	0.8	NA	Glinide	DM
Mother	Wt	Wt	NA	F	NA		NA		NA	NA	None	Non-DM
2												
Proband	p.Gly261Arg	c.-154_-160dup	3	F	110	6.1	5.8	40	NA	NA	None	IGT
Father	Wt	Wt	NA	M	NA		NA		NA	NA	None	Non-DM
Mother	p.Gly261Arg	c.-154_-160dup	18	F	NA		NA		NA	NA	SU→None	DM
Sibling	p.Gly261Arg	c.-154_-160dup	4	M	NA		NA		NA	NA	None	Non-DM
3												
Proband	p.Val182del	p.Gly31Asp	3	NA	NA		6.0	42	1.1	NA	None	DM
4												
Proband	p.Gln290Ter	p.Pro291Arg	7	F	134	7.4	6.3	45	NA	IGT†	None	IGT
Father	p.Gln290Ter	Wt	NA	M	135	7.5	6.3	45	NA	NA	NA	DM
Mother	Wt	p.Pro291Arg	40	F	NA		NA		NA	NA	None	Non-DM
5												
Proband	p.Gln290Ter	p.Pro291Arg	5	M	191	10.6	6.0	42	NA	NA	None	IGT
Father	p.Gln290Ter	p.Pro291Arg	38	M	163	9.1	6.0	42	1.22	NA	None	IGT
Mother	Wt	Wt	NA	F	NA		NA		NA	NA	None	Non-DM
Present study												
Proband	p.Arg191Trp	c.1624-15G>A	10	F	124	6.9	6.9	52	2.78	↑ Glu	None	DM
Father	p.Arg191Trp	Wt	23	M	NA		6.7	50	NA	NA	None	DM
Mother	Wt	c.1624-15G>A	40	F	NA		< 6.0	42	NA	NA	None	Non-DM

*We identified seven cases associated with *GCK* and *HNF1A* variants in four studies. Proband is noted at the top of each reference. Ref.3 had not shown details of the parents; †IGT without insulin resistance; high glucose levels at 2-h.

BMI, body mass index; CPR, C-reactive protein; DM, diabetes mellitus; F, female; Fa, father; *GCK*, glucokinase; Glu, glucose; HbA1c, glycated hemoglobin; *HNF1A*, hepatocyte nuclear factor 1 alpha; IGT, impaired glucose tolerance; IR, insulin resistance; M, male; Mo, mother; NA, not available; OGTT, oral glucose tolerance test; Sib, sibling; SU, sulfonylurea; Wt, wild type.

Variant interpretation is important for predicting clinical phenotypes and elucidating disease mechanisms. The *GCK* p.Arg191Trp allele appears to be a relatively common variant site in MODY2, identified in previous studies reporting *GCK* MODY (9). Other variants located in the 191 position of *GCK* have been suggested to be pathogenic (10), while *GCK* p.Arg191Trp is pathogenic in ClinVar so appears to be causative of diabetes in the patient’s father’s familial history. Consequently, *GCK* p.Arg191Trp is classified as ‘Pathogenic’ (PS1, PM1, PM5, PP1, PP3, PP4) in the American College of Medical Genetics (ACMG) guidelines. It remains unclear whether the identified *HNF1A* c.1624-15G>A variant influences the onset of MODY. This variant was previously reported in a patient with MODY (11) without supporting evidence of impact. In several *in silico* analyses, the pathogenicity of this variant differed across models. Consequently, the *HNF1A* c.1624-15G>A variant was classified as ‘likely benign’ (PP5, BS1, BP2) in the ACMG guidelines. However, the clinical phenotype of *HNF1A* variants is known to be highly variable, even within the same family (12), and has a variety of effects ranging from weak to highly penetrant effects in previous functional studies (13). The genome-wide association studies have identified genetic susceptibility variants for diabetes (14). Therefore, *HNF1A* may represent a phenotype that cannot be explained by the pathogenicity assessment of simple variants alone.

The molecular endocrinological mechanisms of MODY associated with coinheritance are complex and may be explained by the following mechanisms: (i) GCK and HNF1A exist in the same pathway in beta-cell function (15). HNF1A progressively impairs beta-cell functionality, eventually reducing the response to physiological stimuli. This impairment results in a dominant effect on GCK (1). Low-impact variants are known to be involved in the same pathway development of metabolic syndromes reflecting the small effect of the coinheritance of variants, including diabetes, because of the accumulation of burden (16); (ii) The transcripts of HNF1A include the Matched Annotation from the NCBI and EMBL-EBI (MANE) Select transcript ‘NM_000545.5’ and minor transcript ‘NM_001306179.2’. MANE Select transcripts are biologically supported reference sequences that serve as optimal transcripts for clinical variation analysis, capturing nearly all pathogenic variants. However, some alternative protein isoforms are also biologically relevant (17, 18). The complexity of the genetic and molecular landscape of MODY could be dependent on the different transcripts. Further studies are needed to determine the relationship between genetic variants and molecular mechanisms.

Our study has several limitations. First, we focused on known monogenic diabetes genes using an NGS panel, so it was not possible to rule out the influence of variants outside the target of NGS panel analysis. Secondly, we did not conduct a functional analysis of the detected variants. *In vitro*, functional analysis and detailed family studies are needed to clarify the role of this variant. In addition, the *HNF1A* variants were classified as ‘likely benign’ by the ACMG guidelines, which may have identified functional SNPs with limited effects as potentially causative. However, NGS panel analysis can at least resolve previously reported pathogenic variants associated with the diabetes phenotype and can be considered a trigger for careful follow-up clinical investigation.

In conclusion, our case may provide useful information to elucidate the genotype–phenotype correlations of MODY. The future identification of genotypes could be helpful in predicting long-term prognosis in MODY patients.

## Declaration of interest

The authors declare that there is no conflict of interest that could be perceived as prejudicing the impartiality of this case report.

## Funding

This work did not receive any specific grant from any funding agency in the public, commercial, or not-for-profit sector.

## Patient consent

A written informed consent has been obtained from the patient for publication.

## Author contribution statement

DW, HY, and HN were directly involved in the management of the patient. DW drafted the case report. All of the authors contributed to and approved the final draft of the report.

## Patient’s perspective

The patient felt that the genetic analysis associated with MODY was useful in suggesting careful clinical follow-up, which enabled her to understand the need of exercise and alimentary therapy. Her general health and quality of life are better now, so she and her parents were satisfied about the genetic analysis and treatment.
